# Predictive Modelling of Contagious Deforestation in the Brazilian Amazon

**DOI:** 10.1371/journal.pone.0077231

**Published:** 2013-10-18

**Authors:** Isabel M. D. Rosa, Drew Purves, Carlos Souza, Robert M. Ewers

**Affiliations:** 1 Department of Life Sciences, Imperial College of London, Ascot, United Kingdom; 2 Computational Ecology and Environmental Science, Microsoft Research Cambridge, Cambridge, United Kingdom; 3 Imazon – Amazon Institute of People and the Environment, Belém, Pará, Brazil; Cirad, France

## Abstract

Tropical forests are diminishing in extent due primarily to the rapid expansion of agriculture, but the future magnitude and geographical distribution of future tropical deforestation is uncertain. Here, we introduce a dynamic and spatially-explicit model of deforestation that predicts the potential magnitude and spatial pattern of Amazon deforestation. Our model differs from previous models in three ways: (1) it is probabilistic and quantifies uncertainty around predictions and parameters; (2) the overall deforestation rate emerges “bottom up”, as the sum of local-scale deforestation driven by local processes; and (3) deforestation is contagious, such that local deforestation rate increases through time if adjacent locations are deforested. For the scenarios evaluated–pre- and post-PPCDAM (“Plano de Ação para Proteção e Controle do Desmatamento na Amazônia”)–the parameter estimates confirmed that forests near roads and already deforested areas are significantly more likely to be deforested in the near future and less likely in protected areas. Validation tests showed that our model correctly predicted the magnitude and spatial pattern of deforestation that accumulates over time, but that there is very high uncertainty surrounding the exact sequence in which pixels are deforested. The model predicts that under pre-PPCDAM (assuming no change in parameter values due to, for example, changes in government policy), annual deforestation rates would halve between 2050 compared to 2002, although this partly reflects reliance on a static map of the road network. Consistent with other models, under the pre-PPCDAM scenario, states in the south and east of the Brazilian Amazon have a high predicted probability of losing nearly all forest outside of protected areas by 2050. This pattern is less strong in the post-PPCDAM scenario. Contagious spread along roads and through areas lacking formal protection could allow deforestation to reach the core, which is currently experiencing low deforestation rates due to its isolation.

## Introduction

The Amazon is the largest remaining continuous tropical rainforest on Earth. It covers about 6 million square kilometres and crosses nine nations' boundaries. Brazil is the country that hosts the largest portion (about 60% of the area) of the Amazon. This region is characterized by its high cultural and biological diversity[1], but by 2009 already 19% of its forest cover had been converted to other land uses[2]. Deforestation models have been developed to predict which areas are more likely to be deforested in the future and to simulate the impacts of different conservation and market strategies[3], [4], or climatic trajectories and environmental policies[5], on the spatial patterns of future forest cover.

The rate of deforestation – that is, the area deforested per year – in the Brazilian Amazon is highly variable [6]. These fluctuations are related to several factors such as the economic health of the country, infrastructure development, and the world's demand for agricultural products, such as beef or soybeans[6]–[9]. More recently, governance through command and control, restriction to rural credits and expansion of protected areas, helped by a global economic crisis, seem to have contributed to reduce deforestation[10] going in an opposite trend to Brazil's economic growth[11].Although these regional and global factors influence the deforestation rates in the Brazilian Amazon, deforestation is ultimately the sum of thousands of local deforestation events, which occur with an intensity that varies greatly across the region due to many factors including physiographic attributes, access to infrastructure, human population characteristics and dynamics, and socioeconomic organization[12].

Geist and Lambin [13] identified two types of causes for tropical deforestation: proximate causes (*e.g.* infrastructure expansion and agriculture expansion) are human activities that directly lead to change at the local level; and underlying causes, which can be demographic (population dynamics), economic (economic growth or change), technological (improvement or development) or political (environmental laws or policies). When modelling land cover change, modellers aim to select statistically variables that best represent these causes at the scale the model is being developed. For example, economic variables in small-scale models might include the decisions of private actors such as farmers who decide whether they will deforest part of their land [14]–[16].By contrast, larger-scale models (such as the one we present here), cannot address this fine-scale decision-making process and instead focus on what drives deforestation at the regional scale, such as landscape-scale changes in agricultural land and/or infrastructure development plans [4], [17], [18]. However a model may represent the process of deforestation, and whichever predictor variables it may include, it is crucial that the models are constrained against observational data, such that their predictors are at least consistent with the rates and patterns of deforestation observed in the recent past.

The single most important factor that drives deforestation in the Brazilian Amazon is agricultural expansion. Climate and soils are the main constraints to agriculture[19], and infrastructure such as roads determine the ease with which agricultural products can be transported to market. The inaccessibility and humid climate of the northwest regions of the Amazon leaves states like Amazonas less agriculture-prone, whereas along the southern and eastern margins of the Amazon, favourable climates for agriculture combined with extensive road networks explain the concentration of deforestation activity in this particular region of the Amazon[20].

To move beyond these generalizations and to develop policies for managing the balance between agriculture, biodiversity, and carbon storage in the Amazon, requires models that can predict the potential magnitude, timing and spatial patterns of deforestation under different policy scenarios[21]. Several such models, focussing on different aspects of the problem, have been published[3]–[5], [17], [22]. As expected, there are still high uncertainties attached to projecting the location and rate of future deforestation[23], [24] intrinsic to any modelling methodology, mostly because deforestation is statistically rare in the Brazilian Amazon (i.e., the large majority of the forest areas remain unchanged). In addition, uncertainties in predictions arise from differences in the proximate and underlying processes that the models attempt to replicate, and limitation of data in adequate time and space scales. Several models predict the potential spatial pattern of forest cover in the Brazilian Amazon under scenarios that maintain the overall deforestation rate as it is today (business as usual) or scenarios that assume implementation of additional government measures (governance), either for the whole region [3], [4] or for sub-regions Soares-Filho et al. [4]. Additional studies have used more explicit policy scenarios, such as changes in law enforcement [22], or climate change [5],to adjust the regional deforestation rates that drive the models.

Deforestation models use a set of biophysical and socio-economic variables, such as accessibility maps (mainly roads and rivers), landscape maps (land-cover/land-use), cattle and soy prices, human population density and agricultural suitability maps, to predict where deforestation is more likely to occur in the future. Although using different methodologies, they all agree that maintaining the rate of deforestation at current levels would have devastating impacts on the ecosystem and atmosphere, and agree about the relative risk among different regions. Laurance et al. [3]used a simple spatial model to generate two scenarios for the future of the Amazon, with the main difference being the effectiveness of protected areas in preventing deforestation. Both scenarios suggested a dramatic landscape alteration, ranging from 28% to 42% of the region deforested or heavily degraded over the 20 year period beginning in 2001, especially in the south-eastern areas of the Brazilian Amazon. The authors concluded that the efforts to avoid deforestation by improving conservation will be overwhelmed by the destructive trends observed in this region. Soares-Filho et al. [4], although using an improved methodology that allowed for different deforestation rates among the 47sub-regions of the Amazon, found a similar effect, albeit one that took an additional three decades to manifest. All these policy-sensitive scenarios revealed that, given a regional deforestation rate, the spatial pattern will continue to be mostly concentrated in the eastern part of the Amazon where the infrastructures are well developed. Similar results were found by Wassenaar et al. [17], who used the modelling environment CLUE-S to model deforestation in Central and tropical South America until 2010.

Here, we introduce a dynamic and spatially-explicit predictive model of deforestation for the Brazilian Amazon. Our model captures three important aspects of deforestation: uncertainty, emergence, and contagion. The first source of uncertainty is due to deforestation, at the local level, being probabilistic. Because of this stochasticity, we could not expect to predict the details of deforestation perfectly, even if we could predict the magnitude and regional spatial pattern perfectly (by analogy, we could not be expected to predict whether a coin would land heads up or tails up, even if we knew that it was fair). In addition, there is uncertainty in the model structure (*e.g.* best set of predictor variables to use, and how to include their effects), the values of predictor variables (*e.g.* they might be derived, with some error, from satellite images), and in the parameter values of models (*e.g.* the coefficient that determines the impact of a given predictor variable on the probability of deforestation). As such, models predicting deforestation should allow for the calculation of uncertainty on the predicted magnitude, timing and spatial patterns of deforestation resulting from the inherent stochasticity of deforestation events [25]. Although many models in the literature do include stochastic elements in their approaches (e.g. [4], [5]), they do not take advantage of this to provide spatial uncertainty measures associated with their outputs; the uncertainty is only provided by the means of different scenarios. The uncertainty associated with model predictions is crucial to policy makers who need to weigh up the model predictions against other considerations, and other models.

*Emergence* refers to fact that regional or country-wide deforestation rates (or amount of forest loss) are the sum of deforestation occurring at the local scale, influenced by local factors (such as proximity to roads), and local processes. Even regional or global drivers occur via local processes (*e.g.* changes in tax regimes or law enforcement). Because of emergence, the local, and then overall, rates of deforestation can change through time in ways that are not readily anticipated when viewing the phenomenon at the regional scale. Emergence of new deforestation is modelled stochastically but it is driven by local social-economic drivers.

In contrast to previous models, simulating deforestation as an emergent phenomenon allows our model to predict how the overall deforestation rate (or the rate in different regions) might change as deforestation moves across a landscape. This is a fundamentally different approach from accepting the overall rate as a top down input that is imposed upon a spatial model, with that pre-determined amount of deforestation then distributed across the region. This latter approach is widely used by many modelers (e.g.[4], [5], [16]). However, the advantage to our bottom-up approach is that we need to parameterise just one model rather than two: both the top-down and bottom-up approaches need to parameterise spatial models to distribute deforestation across a landscape, but the top-down approach requires a second, separate model to be parameterised to determine how much deforestation will occur.

*Contagion* refers to the fact that location surrounded by recently deforested land, are likely to be more likely to suffer deforestation themselves. This deforestation then increases the probability of other nearby locations. Capturing contagion is crucial because it allows deforestation to spread through space. There is an analogy here with epidemiology[26], [27]: once a disease [deforestation] first invades [begins] in a local region where there are susceptible individuals [forest], it can spread rapidly, especially if there a vector of transport or easy access [roads, rivers, etc.] between infected hosts [deforested areas] and susceptible individuals [forest areas].

Some models in the literature have made use of a ‘patch expander’ function and cellular automata models (*e.g.* [*4*]), which are based on neighbourhood effects and have some similarities to contagion. However, the ‘patch expander’ function is only used post-probability analysis: when calculating the weights of the variables to determine the probability of deforestation, the neighbourhood is accounted for, but as a distance metric (distance to previous deforestation) (*e.g.* [*4*]). Following a ‘seed’ deforestation event, the ‘patch expander’ function is used to create a spatial arrangement that more closely approximates reality, but it depends on pre-determined spatial probabilities of deforestation, rather than influencing those probabilities itself. Further, given that the rate of change is imposed ‘top-down’ in these models, the neighbourhood effect can only influence the location of change, but not the rate of change. By contrast, in our model we embed the neighbourhood effect into the model itself, allowing this contagious process to influence where, and also how much, deforestation will occur. Contagion is also important in the way it combines with stochasticity/uncertainty, because it allows random deforestation to spread into local clusters of deforestation, leading to patterns of deforestation that are very different from those that come from applying deforestation homogenously within regions. Finally, we improve on previous models by conducting a series of stringent model validation tests, comparing the model predictions of one scenario with observed deforestation events over a nine year period.

## Materials and Methods

### Data sources

The first step of the modelling procedure was to identify the main drivers of deforestation in the Brazilian Amazon. The qualitative conclusions from a large literature are that deforestation occurs primarily near previously deforested areas[28], [29], near roads[28], [30]–[33], near markets[28], [29], [34],in areas with a pronounced dry-season[29], [31], and in regions that have been previously logged[35]. Deforestation does occur in protected areas, but the rate tends to be lower than outside protected areas, as is the case in Rondônia [29], [33], [36], [37]. However, in many other parts of the Brazilian Amazon this apparent effect is partially confounded with the fact that protected status tends to be conferred on relatively isolated regions where rates would be expected to be lower anyway [38]. The likely underlying causes of deforestation in the region are changes in gross domestic product (GDP), agricultural GDP, the size of the live cattle herd, and the rate at which temporary and permanent agriculture are expanding [6], [39].

We obtained input data to represent these proximate and underlying causes of deforestation (Table 1). The data was mostly obtained from three Brazilian institutions: Brazilian National Institute for Space Research (INPE), Brazilian Institute for Geography and Statistics (IBGE) and Amazon Institute of People and the Environment (Imazon). It included maps of historical deforestation, forest cover, road networks (official and unofficial), protected areas, rivers, topography, settlements and soil fertility. Dry season length maps were created by applying the methodology developed bySombroek [19] to the historical monthly precipitation data (1960–1990) in the Brazilian Amazon, obtained from the World Meteorological Organization (WMO). Economic data for each of the ∼700 municipalities of the Brazilian Amazon were obtained from IBGE, representing municipality GDP and agricultural GDP, the size of the live cattle herd, and the land area under temporary and permanent agriculture.

**Table 1:** Details of the input data used to calibrate the model for the transition period 2001–2002 and 2009–2010(data name, description, source, reference year and type).

Name	Description	Source	Year	Type
Deforestation	Annual deforestation	INPE^1^	2002 and 2010	Polygon
Previous deforestation	All deforestation occurred	INPE^1^	Until 2001 and 2009	Polygon
Forest cover	Remaining forest cover	INPE^1^	2001 and 2009	Polygon
Roads	Only main rivers	IMAZON^2^	2004 and 2007	Polyline
Rivers	Official and unofficial roads	IBGE^3^	-	Polyline
Settlements	Includes main cities, villages, and smaller settlements	IBGE^3^	-	Points
Topography	Altitude in km	SRTM^4^	-	Raster
Protected areas	Include indigenous lands, federal and state reserves	IMAZON^2^	2001 and 2009	Polygon
Soil Fertility	Reclassified for three classes: low, medium and high	IBGE^3^	-	Polygon
Dry Season Length	Number of months with precipitation <100 mm	WMO^5^	1960/90	Points
Live Cattle	Number of head per municipality (heads)	IBGE^3^	2001/02 and 2009/10	Converted polygon
Temporary Agriculture Area	Total area of temporary agriculture (ha)	IBGE^3^	2001/02 and 2009/10	Converted polygon
Permanent Agriculture Area	Total area of permanent agriculture (ha)	IBGE^3^	2001/02 and 2009/10	Converted polygon
Gross domestic product	Municipalities' gross domestic product	IBGE^3^	2001/02 and 2009/10	Converted polygon
Agricultural gross domestic product	Municipalities' agricultural gross domestic product	IBGE^3^	2001/02 and 2009/10	Converted polygon

1 INPE – Instituto Nacional de Pesquisas Espaciais (http://www.dpi.inpe.br/prodesdigital/prodes.php).

2 IMAZON – Instituto do Homem e Meio Ambiente da Amazônia (http://www.imazon.org.br/).

3 IBGE – Instituto Brasileiro de Geografia e Estatstica (http://www.ibge.gov.br/home/download/geociencias.shtm).

4 SRTM – The Shuttle Radar Topography Mission from National Aeronautics and Space Administration (NASA http://www2.jpl.nasa.gov/srtm/).

5 WMO – World Meteorological Organization (http://www.agteca.com/climate.htm).

The data were separated into two categories of variables: static and dynamic variables[40]. Static variables represented features that are assumed to stay constant through time such as soil fertility, topography, main rivers, state and dry season length. In our model, we also assumed that the distribution of protected areas is a static feature of the region, although the last decade experienced rapid and large expansion of protected areas. Dynamic variables, by contrast, represent features that change through time. In the model we present here, only forest cover itself and the proportion of deforested neighbour cells were treated as dynamic. There are several variables that are dynamic but which we considered to be static in our model due to a lack of data. This includes the economic variables (GDP, cattle herd, area of temporary and permanent agriculture), as we do not have economic models available to predict the value these variables take in the future. Similarly, we considered the road network to be a static feature of the landscape. Note that road networks in the Brazilian Amazon are known to be expanding [41], suggesting they should be considered as dynamic rather than static variables. However, in the absence of a validated model of road network expansion, we were unable to replicate this process and hence treated road networks as a static landscape feature (see Discussion). Static variables were calculated just once, in the beginning of the modelling process, whereas dynamic variables were re-calculated at each time step (year). Model variables were estimated individually for 5×5 km pixels across the Brazilian Amazon.

In contrast to previous modelling, we used as metric of local deforestation the proportion of deforested grid-cells in the neighbourhood of the focal cell. This contrasts with the usual approach of using Euclidean distance to the closest deforested cell[3]–[5]. We made this decision because our model updates the local deforestation probabilities as the neighbourhoods change through time. Within this dynamic framework, the Euclidean distance metric results in a very rapid, but diffuse, expansion of deforestation, characterized by rapidly spreading regions, within which there are a few deforested cells within a matrix of intact forest. This diffuse pattern of deforestation is not apparent in current deforested landscapes. The rapid expansion in models occurs because deforestation in a single cell immediately reduces the Euclidean distance over a large neighbourhood around that cell (in fact all cells, anywhere in the whole region, which are closer to the new deforestation event than to any previous event, experience an increase in deforestation probability). The diffuse pattern occurs because, if a cell already has a single deforested cell nearby, further deforestation events can have no effect on the local rate of deforestation (any event further away than the closest previous event has no further effect on local deforestation). By contrast, when using neighbourhood metric such as employed here, the local deforestation probability responds only to neighbouring cells, and builds continuously as the surrounding neighbourhood is deforested. As a result, simulations using the neighbourhood metric result in deforestation spreading in a well defined front, characterized in space as a rapid gradient from intact forest, to almost pure deforestation – a pattern that is consistent with observed patterns of deforested land.

### Model structure, parameterization and selection

**Figure 1:**
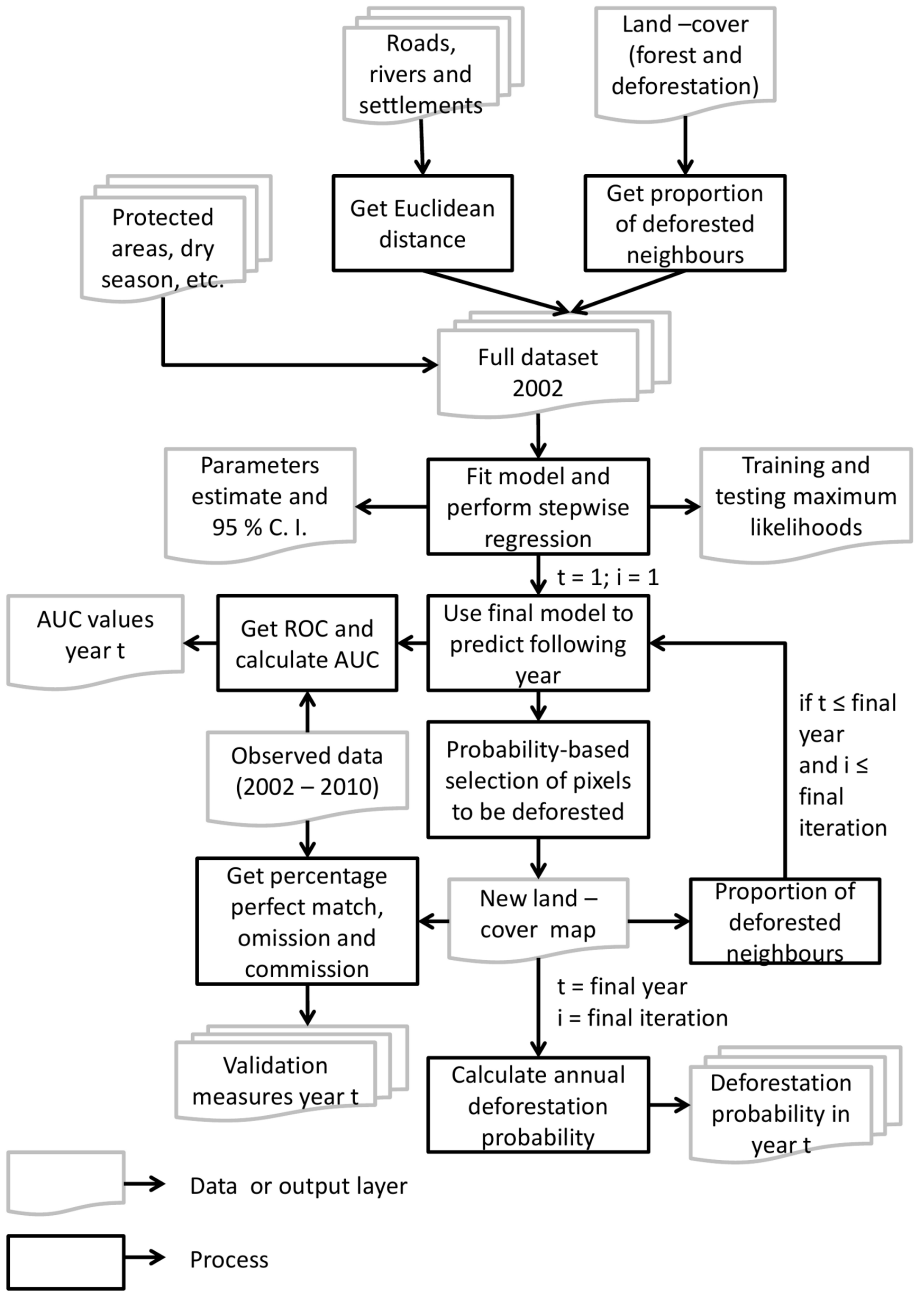
Modelling procedure flowchart. The flowchart illustrates the construction and running of the deforestation model. *i* is the model iteration, *t* is the year, ROC refers to the Receiver Operating Characteristic and AUC is the area under the ROC curve.

Our model is based around *P_defor,x,t_*, the probability that cell *x* becomes deforested in a set interval of time *t*. The fact that *P_defor,x,t_* is specific to a given time *t* illustrates how our model updates the local deforestation through time. We defined this probability as a logistic function: 
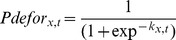
(1)such that as *k_x,t_* goes from minus infinity to plus infinity, *P_defor,x,t_* goes from 0 to 1. We could then write simple linear models for *k_x,t_* as a function of the driver variables affecting location *x* at time *t*. Similar logistic regression techniques have been successfully used and have become the standard method for assessing deforestation probabilities [29], [40], [42]. The modelling procedure flowchart is shown in Figure 1 and the full model C^++^ code is provided in online (Supporting Information S1).

In 2004, the Brazilian government implemented the “Plano de Ação para Proteção e Controle do Desmatamento na Amazônia” (PPCDAM), which implemented a set of enforcement measures to fight illegal deforestation[43]. The PPCDAM also coincided with a global economic downturn that reduced the economic incentives for deforestation[10], and thus the post-2004 period represents a deforestation ‘regime’ that is very different to what was observed before. Before 2004, deforestation rates were increasing rapidly and deforestation was spreading quickly, whereas after 2004 the situation changed and the rates slowed down significantly[10]. Therefore, we fitted models to the data for observed deforestation that occurred pre-PPCDAM, between 2001 and 2002, whereas for a post-PPCDAM scenario the transition year used was 2009–2010.These two time periods represent very different deforestation regimes in the region, and thus the two calibrations of the model should result in different scenarios of deforestation by 2050.

We fitted 106 models to the observed deforestation patterns in each of the two transitions periods representing our two deforestation scenarios. Models differed only in the combination of static and dynamic variables included in the definition of *k_x,t_*. To fit each of these 106 models we used ‘Filzbach’, a freely available C library developed by DP and others (http://research.microsoft.com/en-us/projects/filzbach/). Filzbach uses Markov Chain Monte Carlo (MCMC) sampling techniques to return, for each parameter, a posterior probability distribution from which we can extract the posterior mean, and a credible interval, given the model structure and the data. To carry out the parameter estimation, all that is necessary is to define the log-likelihood, which is a measure of goodness of fit between the model predictions and the observations, given a particular combination of parameters θ: 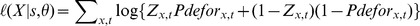
(2)where *Z_x,t_* is the observed deforestation at location *x* at time *t*, and *s* refers to one of the 106 models that we considered. The likelihood defined in Eq. 2 assumes independence among the samples, and is the same likelihood that underlies any standard logistic regression.

The exact set of 106 models was arrived at by forward stepwise regression. We chose the forward stepwise method, widely used in predictive studies[29], [44], [45] to first assess the impact of each variable on the probability of deforestation individually, and then to determine the additional predictive power gained by adding additional variables. In the first step, only the intercept is included, giving a one-parameter model. To assess this model, we used cross-validation, a statistical technique used to assess how accurately the model will predict data that was not used to train the model [46]. The cross validation was carried out by parameterising the model against a randomly selected subset of 50% of locations, then calculating the likelihood of the remaining 50% of the locations, using eq. 2. The purpose of the cross validation was to find a model that included those only predictor variables that had demonstrable predictive ability. Cross validation, where possible, is superior to model selection using information criteria such as the AIC, which is known to often lead to over-fitting [47], [48]. Next, each of the nine variables were added individually to the intercept-only model, creating a set of 2-parameter models that were again assessed with cross-validation. When all two-parameter models were trained and tested, the variable responsible for the highest maximum likelihood model was kept in the model and the remaining eight variables were again added individually. This procedure was repeated until all models were trained and tested, which resulted in a table with each model and the corresponding training and testing likelihoods, from which we selected the ‘best model’ as the one with the maximum test likelihood from all of the 106 models. However, after this procedure was complete, we found that some of the variables included in the best model had a non-significant confidence interval. In these cases, we chose the second best model, which had a slightly lower maximum likelihood, where all variables were in fact significant. This last, conservative, step, was carried out to further reduce the potential for over-fitting. The forward stepwise procedure was repeated for each of the two transition periods corresponding to the pre- and post-PPCDAM scenarios.

### Simulations

Once we had settled on the best statistical model for explaining past deforestation during each of the two transition periods, we used it to simulate future deforestation up to 2050 under the pre- and post-PPCDAM scenarios. To do so, all that was necessary was to re-apply eq. 1 in each time step, recalculating the dynamic variables (e.g. fractional deforestation around each location *x*), and using a slightly different set of parameter values at each iteration (to incorporate parameter uncertainty), drawn from a Gaussian distribution using the estimated mean and standard deviation for each parameter. This provided an updated *P_defor,x,t_* for each location *x*, which was then deforested with that probability. In practise, this was implemented as follows: for each *x*, draw a random number from a uniform distribution bounded at 0 and 1, and deforest *x* if this number is less than *P_defor,x,t_*. After these deforestation events were implemented, *P_defor,x,t_* was calculated for every location *x* again, allowing for another round of deforestation. This procedure illustrates the three key aspects of our model mentioned above (see Introduction).The model is stochastic, because each individual deforestation event is drawn randomly using a weighted probability. Deforestation is contagious, because deforestation at location *x* increases the probability of deforestation at neighbouring locations, inducing further deforestation events which themselves further increase the probability of deforestation in the neighbours of the neighbours. Finally, the total (region-wide) deforestation rate at any time *t*, emerges as the sum of the local, stochastically determined, deforestation events, rather than being imposed top-down. The total deforestation rate can also vary through time, due to changes in the spatial configuration of forest cover in relation to the static and dynamic variables incorporated in the model. During each simulation, we kept a record of the fraction of cells undergoing deforestation at each time step, and a record of the pattern of forest vs. non-forest at each time step.

To calculate the uncertainty in model predictions, we ran the simulations multiple times (*N* = 100iterations) and summarised the outputs across models at each time step. This allowed us to construct confidence intervals around our model predictions, rather than providing a single ‘answer’. The uncertainty is represented by our final simulation outputs are a deforestation probability map calculated for each year in the simulation as the number of times a pixel was selected to be deforested in that year, divided by the total number of iterations. For each pixel that was deforested in the simulations, we also estimate the mean date at which it was deforested as well as the inter-quartile range around that date. This quantifies the uncertainty in the exact timing of deforestation events in the model simulations.

### Model validation

We validated our model predictions for the pre-PPCDAM scenario (parameterised with the transition year 2001–2002) against observed data for each year within the period 2002–2010 by calculating the area under the Receiver Operating Characteristic (ROC) curve in each year, which is used in many land-cover change studies [17], [49], [50]. For the post-PPCDAM scenario (parameterised with the transition year 2009–2010), the validation was only done for the first year of predictions (2010), reflecting the time period of deforestation data used to calibrate out model. For each of the 100 model iterations, we calculated the area under the ROC curve (AUC) value and three measures of precision on a pixel-by-pixel comparison: perfect match (the model predicts the exact location of deforestation), commission (over-predicting, the model predicts deforestation events that did not happen) and omission (under-predicting, the model did not predict deforestation in a location where deforestation happened). These three measures were calculated annually, using the observed and predicted annual deforestation maps; and for the pre-PPCDAM scenario were also calculated cumulatively, using the observed and predicted sum of deforestation at each time step (2002, 2002–2003, 2002–2004, etc.), for the time period from 2002 through 2010. Additionally, we calculated the proportion of observed annual and cumulative deforestation that occurred within certain distances (0, 5, 10, 25 and 50 km) of our predicted deforestation at each time step.

## Results

### Model calibration

**Figure 2:**
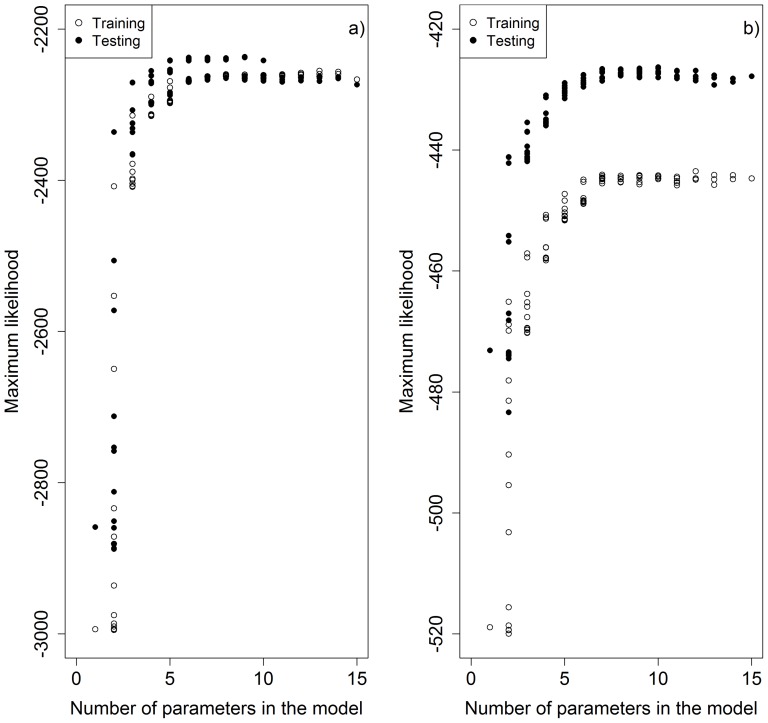
Stepwise regression output. Figure shows both training and testing maximum likelihoods achieved by each of the 106 models used to explain deforestation rates in the Brazilian Amazon in the (a) pre-PPCDAM and (b) post-PPCDAM scenarios.

In both scenarios, the test likelihood returned from the cross validation increased rapidly with the addition of the first parameter, with additional parameters having progressively smaller impact on predictive power (Fig. 2). Most variables were found to have the impact we expected on deforestation probabilities (Table 2). For instance, for both periods, distance to roads or rivers or settlements had a negative sign suggesting higher deforestation closer to these features. Also, protected areas had a negative sign, but here representing a lower probability of deforestation inside these areas when compared to unprotected land. Further, annual increases in GDP, the size of the live cattle herd, and the area of land in permanent agriculture were found to have a positive impact on the probability of deforestation. By contrast, change in temporary agriculture area was non-significant in both cases, whereas change in agricultural GDP was found to only be significant in the period post-PPCDAM.

**Table 2:** Mean and 95% confidence intervals of the single variable models, for each scenario (pre- and post-PPCDAM).

	Pre-PPCDAM			Post-PPCDAM		
Parameter	Mean	Lower	Upper	Mean	Lower	Upper
name		limit	limit		limit	limit
Intercept	−4.7911	−4.8830	−4.6983	−5.9828	−5.9997	−5.9372
Previous Deforestation	4.9659	4.5774	5.2429	2.1113	−0.8230	3.2692
Roads	−0.00018	−0.00021	−0.00016	−0.00011	−0.00018	−0.0001
Rivers	−0.00002	−0.00003	−0.00001	−0.00005	−0.00007	−0.00003
Settlements	−0.00005	−0.00005	−0.00004	−0.00006	−0.00009	−0.00004
Topography	−1.9345	−1.9991	−1.7693	−1.8200	−1.9969	−1.5012
Soil fertility	0.0001	0.0000	0.0005	0.0002	−0.0002	0.0005
Dry season length	0.3194	0.2931	0.3394	−0.3075	−0.4019	−0.2190
Cattle	0.3947	0.1890	0.5294	0.1388	0.0013	0.3916
GDP	0.0532	0.0007	0.1535	0.0182	0.0005	0.0566
GDP agro	0.0064	−0.0002	0.0201	0.0447	0.0013	0.1882
Temporary Agriculture	0.0273	−0.0001	0.0849	0.0119	−0.0004	0.0413
Permanent Agriculture	0.3687	0.1534	0.5828	0.0658	0.0006	0.1966
Prot. Areas–State	−0.6281	−1.1474	−0.1856	−1.7402	−1.9976	−1.2311
Prot. Areas–Federal	−1.0426	−1.7319	−0.6574	−0.5949	−1.4540	0.0360
Prot.Areas–Indigenous	−0.9151	−1.1168	−0.7429	−0.3833	−0.8982	−0.1657
State – Maranhao	−0.4757	−1.2139	0.2250	0.3687	−1.2280	1.6955
State – Tocantins	−0.4989	−1.6543	−0.0464	0.2303	−1.6190	1.0590
State – Pará	−0.5673	−0.7502	−0.3772	−0.6250	−1.1601	−0.2796
State – Roraima	−0.9969	−1.3064	−0.7918	−0.3807	−1.0302	0.0041
State – Amapá	−0.8455	−1.3031	−0.5581	−1.1259	−1.9959	−0.3580
State – Acre	−0.3752	−0.5039	−0.2386	−1.1043	−1.8357	−0.4237
State – Rondônia	−0.1172	−0.2069	−0.0410	−0.3711	−1.0441	−0.0691
State – Amazonas	−0.5297	−0.6677	−0.4354	−0.4841	−1.0160	−0.2883
State – MatoGrosso	−0.0590	−0.1149	−0.0005	−0.3109	−1.1723	−0.0439

The most important variable was distance to roads, followed in turn by neighbourhood deforestation and protected areas, with our analysis indicating that deforestation probabilities were lowest in Indigenous lands, slightly higher in Federal and then State reserves, and highest in unprotected land. State also exerted considerable influence on deforestation probabilities and was retained in our best model. The only difference between scenarios, apart from variations in the effect size of individual variables (Table 3), was the inclusion of total GDP in the post-PPCDAM scenario. Adding additional variables had negligible effects on the test likelihood (Fig. 2) and were consequently omitted from the final model. The final set of parameter values used in the deforestation simulation for each scenario are shown in Table 3.

**Table 3:** Mean and 95% confidence intervals of the final set of parameter inputs used in the deforestation simulations, for each scenario (pre- and post-PPCDAM).

	Pre-PPCDAM			Post-PPCDAM		
Parameter name	Mean	Lower limit	Upper limit	Mean	Lower limit	Upper limit
Intercept	−3.70	−4.88	−2.57	−4.96	−5.92	−3.28
Previous Deforestation	2.10	1.70	2.57	2.32	1.61	3.07
Roads	−0.00011	−0.00013	−0.00009	−0.00006	−0.00009	−0.00003
GDP	-	-	-	0.45	0.08	0.76
Prot. Areas–State	−0.18	−0.73	0.09	−0.18	−0.97	0.52
Prot. Areas–Federal	−0.40	−0.84	−0.19	−0.71	−1.25	−0.19
Prot.Areas–Indigenous	−0.52	−0.75	−0.41	−0.42	−0.75	−0.13
State – Maranhao	0.10	−0.25	0.451	0.63	−1.21	1.91
State – Tocantins	−0.40	−0.58	−0.23	−0.04	−1.34	0.90
State – Pará	−0.11	−0.2	0.02	0.01	−0.53	0.43
State – Roraima	−0.49	−0.6	−0.4	−0.09	−0.64	0.31
State – Amapá	−0.31	−0.4	−0.2	−0.30	−0.96	0.20
State – Acre	−0.10	−0.2	−0.04	−0.09	−0.41	0.17
State – Rondônia	−0.01	−0.1	0.04	−0.08	−0.34	0.12
State – Amazonas	−0.18	−0.2	−0.1	−0.19	−0.43	−0.01
State – MatoGrosso	0.00	−0.04	0.04	−0.1	−0.29	0.04

At each iteration, a slightly different set of parameters' values is drawn from these distributions to be used in the model that predicts deforestation from 2002 (or 2010 in the post-PPCDAM scenario) to 2050.

### Model validation

In both the pre- and post-PPCDAM scenarios, the models had apparently strong predictive power with mean AUC values of 0.92 in the first year. In the pre-PPCDAM scenario, which had a longer period of model validation, we found that the predictive power of the calibrated parameter set declined through time to 0.86 over the first 8 years of model predictions (2002–2010).

**Figure 3:**
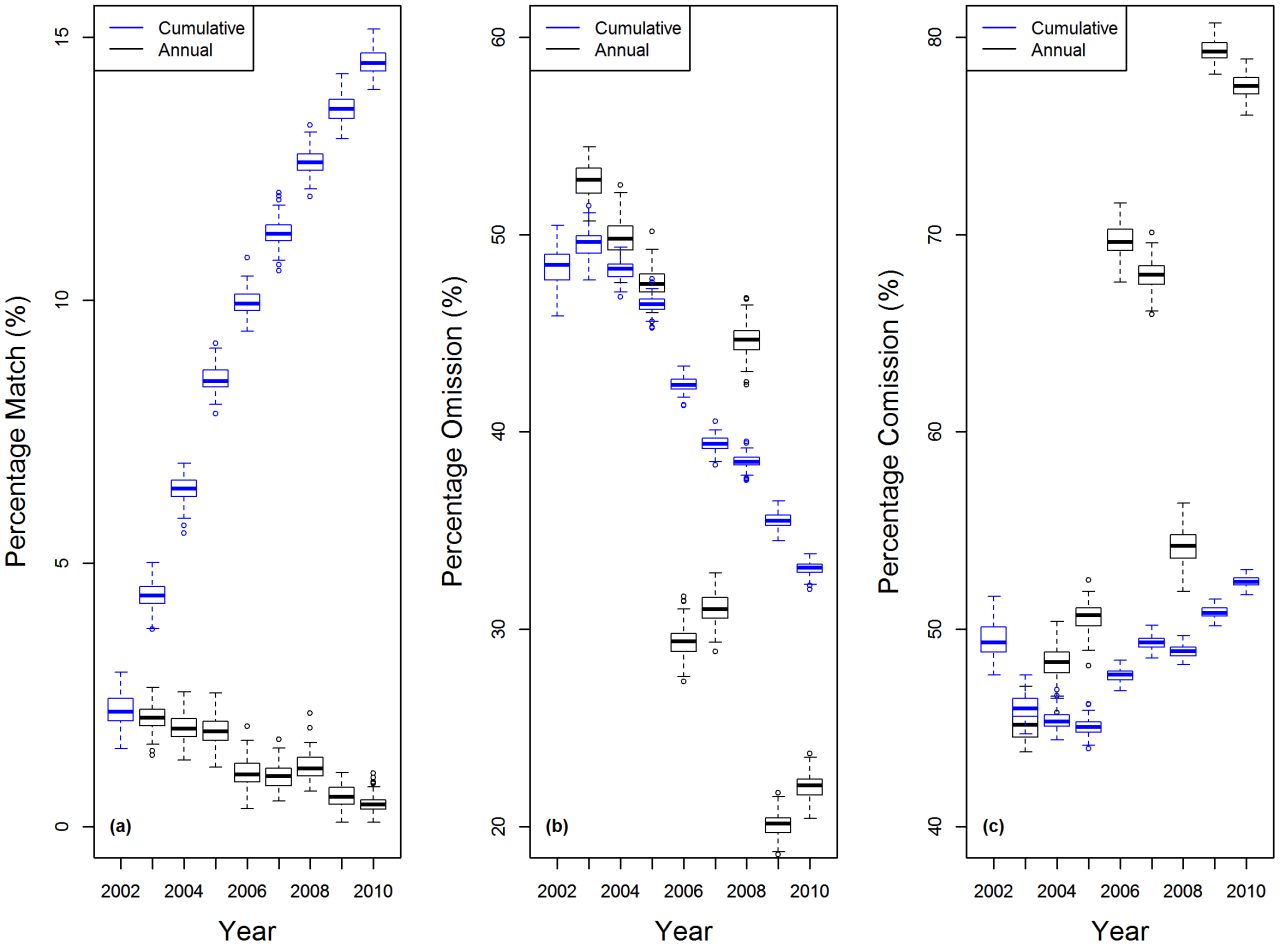
Pre-PPCDAM model validation results comparing pixel by pixel predicted and observed deforestation between 2002 and 2010. Three validation statistics are presented: (a) mean percent of perfect match; (b)errors of omission; and (c)errors of commission. Validations were conducted in two ways. The ‘annual’ validations compare predictions from a single year with observations for that same year, whereas the‘cumulative’ validations compare all deforestation predictions up to and including that year with observations of cumulative deforestation over the same time period. Variation in these values arises from the 100 model iterations.

**Figure 4:**
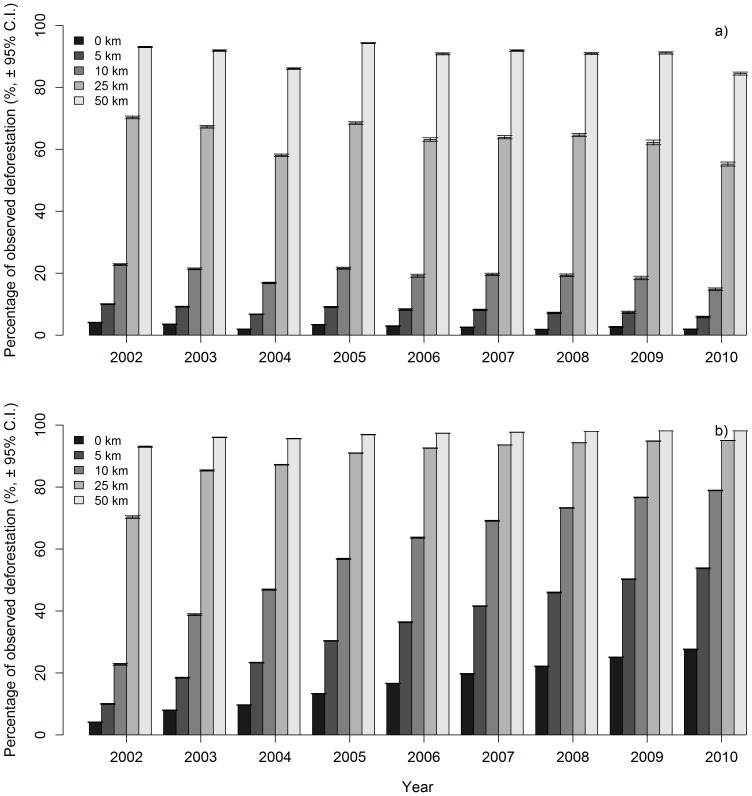
Pre-PPCDAM model validation showing the spatial dependence of model accuracy. Values represent the proportion of (a) annual and (b) cumulative observed deforestation from 2002 through 2010 that fell within a threshold distance from predicted deforestation.

In addition, for the pre-PPCDAM scenario, we compared the model predictions with observed data pixel by pixel, both annually and cumulatively from 2002 through 2010. Although most land cover change modellers prefer to compute statistics that compare model outputs with those from a random distribution, such as the Kappa-family of metrics[4], [5], we believe that making more demanding pixel-by-pixel comparisons is a more informative and more direct representation of how accurately the model predicts the actual rate and spatial patterns of deforestation[51]. On an annual basis, the model predictions perfectly matched an average of just 2% of observed deforestation events in 2002 and this percentage dropped further through time (Fig. 3a). However, the cumulative prediction accuracy improved through time, for the time span that validation data is available, with an average of 15% perfect match between predicted and observed deforestation by 2010 (Fig. 3a).These results suggest that the model is correctly predicting the general spatial pattern of deforestation, but that the ability to predict the exact sequence of deforestation events is very poor. The majority (>60%) of all annual observed deforestation fell within 25 km of predicted deforestation, and virtually all observed deforestation (84 to 94%) was within 50 km of predicted deforestation. There was no significant change through time in these values (Fig. 4a). When analysed cumulatively (Fig. 4b), an average of 80% of all observed deforestation from 2002 through 2010 occurred within 10 km (2 pixels) of deforestation predicted by the model.

Annual rates of omission errors closely tracked the rate of deforestation (Fig. 3b), with the model omitting more deforestation events in years with more deforestation and omitting fewer deforestation events in years with less deforestation. Commission errors were high and followed the opposite pattern, with most pixels that were predicted to be deforested in a particular year not being observed (Fig. 3c). When validated against cumulative patterns of predicted and observed deforestation commission errors increased through time whereas omission errors decreased, again indicating that the emergent spatial patterns of deforestation are reliable but that the exact sequence in which pixels and deforested is poorly predicted (Fig. 3b and c).

### Rate and location of land-cover change

**Figure 5:**
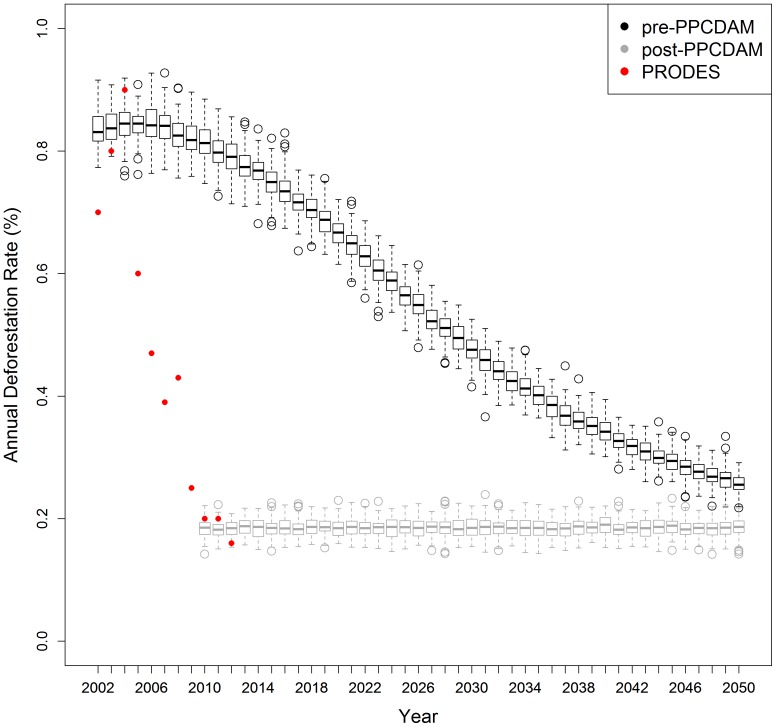
Predicted deforestation rate in the Brazilian Amazon between 2002 and 2050. Deforestation rates emerged from the local deforestation probabilities in the spatial model, for both the pre- and post-PPCDAM scenarios, and variation in these values arises from the 100 model iterations. Thick lines represent the median, boxes the inter-quartile range and whiskers the maximum and minimum simulated deforestation rates.

The total amount (or rate) of deforestation in any given year emerged bottom-up from the accumulation of stochastically determined local deforestation events, and predicted that deforestation rates would almost halve by the year 2050 under the pre-PPCDAM scenario (Fig. 5). Annual differences in deforestation rates among model iterations of the pre-PPCDAM scenario were as much as 0.2%, whereas the post-PPCDAM scenario showed a more stable rate through time (Fig. 5). In 2002, our first year of model predictions for the pre-PPCDAM scenario, the model predicted an average deforestation rate of 0.85%, and for 2010 the pre-PPCDAM predicted a deforestation rate of 0.82% whereas under the post-PPCDAM scenario the average was just 0.2%. Model predictions from the first three years of simulations in both pre- and post- PPCDAM scenarios were in line with observations for this region from INPE [52].These results suggest that the Brazilian government's PPCDAM program, helped by the coincident global economic downturn, seems to have been successful in lowering deforestation rates. Because we only have one dynamic variable in the model (deforestation neighbourhood), the predicted deforestation rate dropped almost constantly through time in the pre-PPCDAM scenario, presumably because all pixels near roads that had the highest deforestation probabilities became deforested leaving behind just pixels with relatively low deforestation probabilities. This pattern is less evident in the post-PPCDAM scenario since the predicted rate of deforestation is much lower. Between the years 2010-2050, the difference in deforestation rates between the pre- and post-PPCDAM scenarios suggests that implementation of PPCDAM will have resulted in an average cumulative reduction in deforestation of 389,884 (±657, 95% C.I.) km^2^.

**Figure 6:**
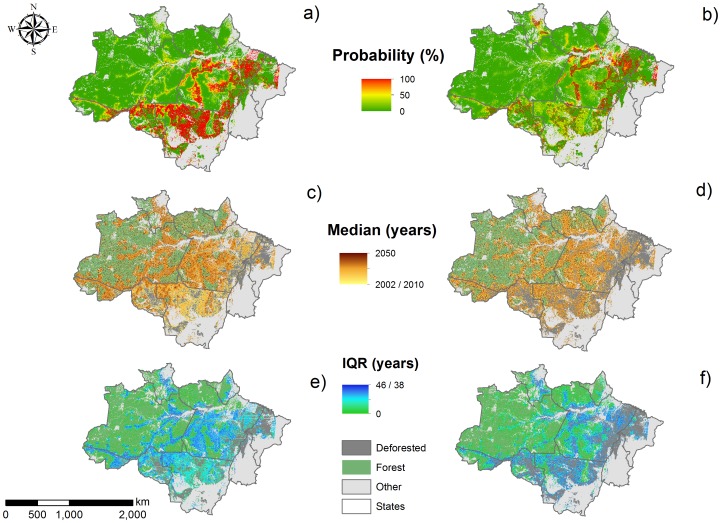
Deforestation predictions for the Brazilian Amazon under the pre- and post-PPCDAM scenarios. Cumulative deforestation probability in the year2050 (a) under the pre-PPCDAM and (b) the post-PPCDAM scenario; the wave of deforestation, represented as the median year in which each pixel was deforested (c) under the pre-PPCDAM and (d) the post-PPCDAM scenario; and uncertainty in the model predictions, quantified as the inter-quantile range of the year in which each pixel was deforested (e) under the pre-PPCDAM and (f) the post-PPCDAM scenario. In panels (c,d) and (e,f), measures of central tendency and variation were obtained by comparing model outputs from the 100 model iterations.

Using the annual deforestation probability outputs (Supporting Information S2), we mapped the cumulative deforestation probability predicted for 2050 for both scenarios (Fig. 6a,b), and created two video outputs showing deforestation probability accumulating from 2002 (or 2010 if post-PPCDAM) to 2050 (Supporting Information S3).We found that pixels within a short distance from roads had a very high probability of becoming deforested in the next 40 years, but that protected areas play a vital role of inhibiting the spatial expansion of deforestation.

The “wave”, or temporal sequence, of deforestation across the Brazilian Amazon (Fig. 6c,d) suggests that the sequence of deforestation events follows the deforestation probabilities themselves for both scenarios, with deforestation occurring first along the southern and eastern boundaries of the Amazon before spreading along and out from major highways that penetrate the Basin. However, the magnitude of change is much less intense in the post-PPCDAM scenario. Each iteration of our model represented a different possible future, because we allowed for uncertainty in the model parameters and stochastically determined deforestation events, meaning that in different iterations pixels could be deforested in different years. To capture this uncertainty in our predictions of the wave of deforestation, we mapped out a measure of variance, the inter-quartile range, around our estimates of the year in which each pixel was deforested. The median value of the inter-quartile ranges was 15 years, showing a large amount of uncertainty in the exact timing of deforestation events. In general, for both scenarios, model uncertainty was lowest along the Arc of Deforestation and in areas where nearby roads give immediate access to forest. In the most inaccessible parts of the Brazilian Amazon, the inter-quartile range around our deforestation predictions was as high as 40 years.

## Discussion

With a predicted increase in global human population and, consequently, a rise in external demand for agricultural products, the future of the Brazilian Amazon is at stake if pre-PPCAAM annual deforestation rates prevail in the next decades. In order to predict the potential impacts of deforestation on biodiversity and evaluate the potential effectiveness of conservation strategies, it is vital to be able to accurately predict the magnitude and geographical distributions of future deforestation. However, as we showed the models are not yet accurate enough and it remains vital to improve the prediction of deforestation models. Our model is not the first attempt to make these predictions, but goes beyond previous attempts by capturing three important aspects of deforestation which have poorly been explored in the past: uncertainty, emergence, and contagion. Additionally, we use the stochastic nature of the model to specifically estimate uncertainty around model predictions, which we found to be substantial, by allowing model parameters to vary at each model iteration. Overall our analyses suggest that we can have some confidence in the spatial patterns of cumulative deforestation that will emerge over the coming decades, but that we have little, if any, power to predict the exact sequence of deforestation events at the level of individual pixels. This remains one of the biggest difficulties in land cover change models [53].

Our statistical analysis identified several predictor variables that had demonstrable predictive power for deforestation, but also showed that adding extra predictor variables and parameters to the model does not necessarily lead to a better model. We found that, for both scenarios, the proportion of deforested neighbours has a strong influence on the probability of a given pixel itself being deforested events, which indicates that the “behaviour” of deforestation mimics that of an infectious disease, increasing our confidence in modelling deforestation as a contagious process. Once it starts in a region it can spread very rapidly [49], [54] and that spread is even more rapid when roads provide easy access to forests.

Roads play a major role in determining where and how much deforestation will occur and our model has confirmed a large body of literature that emphasises the important impact of roads on deforestation patterns [13], [55]. Regrettably, there is presently a lack of validated models predicting the rate and pattern of expansion of the road network itself in the Amazon [56], making it difficult to include them as a dynamic variable in a deforestation model [4]. We can estimate where and when official roads will be created[4], but the same is not true for unofficial roads which are very widespread in the region and represent a major threat to forests [41].Given that we allowed the rate of deforestation to emerge from the model itself in a bottom-up manner for both our scenarios, the fact that the predicted deforestation rate drops through time in the pre-PPCDAM is partly, perhaps mostly, an artefact of having a static road network as an input variable in the model, although we know the road network is continuously expanding in this region. We would expect that incorporating a dynamic road network in the model[56], which would continually expand roads through time, would keep deforestation probabilities high and lead to a more steady, or even increasing (as forest would decrease), deforestation rate for both scenarios. However, we also note that economic models of deforestation in the Brazilian Amazon predicted deforestation rates to begin declining under a Business as Usual scenario around 2030 [4], although the reduction predicted there was much lower than predicted by our model. The influence of using static road maps in our model predictions ensures that our predictions of the difference in cumulative deforestation between the pre- and post-PPCDAM scenarios can be considered conservative.

Protected areas strongly constrained the spatial pattern of deforestation in the pre-PPCDAM and post-PPCDAM scenarios, in line with other more direct analyses of the effectiveness of Amazonian protected areas[36], and we also found that different types of protected areas exert stronger or weaker limits of deforestation [36]. Where roads were adjacent to protected areas, we found that deforestation was much more intensive relative to the wider spatial spread of deforestation that occurred around road networks that did not abut protected areas. This suggests, therefore, that the implementation of reserves bounding roads should be supported as an effective means of limiting the spatial spread of deforestation [37].

Within the nine states of the Brazilian Amazon, Mato Grosso, Pará and Rondônia are the three that have had the highest levels of annual deforestation [29] and this will remain true for the foreseeable future (although there are some early signs of deforestation starting to reach the lower part of the Amazonas state) in both our scenarios, unless governance is improved and there is a strong incentive to restore already degraded lands. According to our pre-PPCDAM simulations, by 2050 Mato Grosso and Rondônia will virtually have no forest left outside the reserves, and the reserves themselves will become very isolated, even though our pre-PPCDAM scenario shows a clear decline of deforestation rates. This pattern is less strong in the post-PPCDAM scenario due to the lower rate of deforestation; however, even here the same states will have the strongest landscape modification. By contrast, even in our most aggressive scenario of deforestation (pre-PPCDAM), Amazonas and Roraima are protected by their inaccessibility, benefitting from the ‘passive protection’ that arises from their geographic isolation[38], [57]. However, new planned initiatives to pave roads in all Brazilian Amazon means deforestation will continue to progress, and in particular, in these states[4] the passive protection will be significantly reduced.

Simulation results show that calibrating our model in a different transition year can have a great impact on the rate and location of predicted deforestation. We exploited this variation by calibrating the model for transitions before and after the implementation of PPCDAM, a strong plan to prevent deforestation by the Brazilian government. Our simulations showed a less aggressive scenario of future deforestation both in terms of rate and spatial spread when the model is fitted after the PPCDAM was implemented, when compared to the pre-PPCDAM simulations which were achieved by calibrating the model for a year before the PPCDAM. If we assume that the conditions post-PPCDAM are maintained into the future, we predict this will nearly 390,000 (±660, 95% C.I.) km^2^ of cumulative deforestation by 2050. However, recent changes to the Brazilian Forest Code suggest that the strong level of reduction in deforestation rates between 2004–2010 may not be maintained into the future[58], although it remains unlikely that rates will climb back to the high values that occurred in the early 2000s.

For both scenarios, because the spatial and temporal patterns of deforestation resulted from stochastic iterations, which also incorporated variation in the model parameters, we were able to capture the temporal and spatial uncertainty in our model predictions. Furthermore, the choice of calibrating our pre-PPCDAM model for the year 2001–2002 enabled us to validate our model predictions for eight consecutive years (2002–2010) both annually and cumulatively, which is rarely done in land-cover change modelling studies. These particular aspects of the model (uncertainty resulting from stochasticity and parameter uncertainty; emergence; contagion; and validation over more than one time step), which have not previously been used together in land-cover change models, allow us to place more confidence on the long term deforestation predictions. The implications from our uncertainty analyses and stringent validations are that we can be more confident in the cumulative pattern of deforestation probabilities through time rather than the exact temporal sequence. However, we are far from accurately predicting the exact sequence in which forests (pixels) will be deforested.

The modelling procedure we have presented here for the Brazilian Amazon under two different scenarios (pre- and post-PPCDAM) can also be used to test the potential impacts of different scenarios, such as the impacts of the construction of new roads and new hydroelectric dams, the implementation of new protected areas, or to estimate biodiversity and carbon losses due to land-cover change. For instance, under the pre-PPCDAM scenario the model predicts rates higher than those observed after the downturn of agriculture in 2005–2006, which is believed to greatly influence the reduction of deforestation in this region [33]. If, however, this economic downturn had translated into less road development the model could dynamically update the road layer and rates would slow down given that the access to forest was stabilizing. Once we re-calibrate the model for the post-PPCDAM scenario using data from 2009–2010, the predicted rates changed considerably and again matched those directly observed by PRODES. However, the largest changes observed in predicted rates between the two scenarios are more directly related to calibration data rather than the emergency property of the model. Therefore, predicting deforestation rates remains the greatest challenge in land cover change modelling. The scenarios presented here were mainly to show the potential of our model structure to quantify the impact of different scenarios, and demonstrate that it can be adapted to address questions about the impacts of policy decisions. Furthermore, tools such as this have potential to be integrated into decision-making processes, providing guidance to conservationists and policy-makers as they plan and test competing land cover decisions. However, these must take into account both the spatial and temporal scales where the model was built and tested, and how the uncertainty in the model output varies at each scale. For instance, models not only can be used to project future trends of deforestation but also to evaluate policy impacts in a long term. However, given that our results showed a low ability to predict the exact temporal sequence of deforestation, we stress the idea that models should provide their users a measure of uncertainty attached to their predictions.We believe that the probabilistic approach we have developed here represents an important step towards the goal of more fully engaging land cover change models with land cover planning decisions.

## Supporting Information

Supporting Information S1 **Commented C++ model code, showing the full model structure, parameterization, selection, and how simulations were performed for one of the scenarios.**
(CPP)

Supporting Information S2 **Annual model outputs (Ascii files) showing the cumulative deforestation probability from 2002 through 2050 (pre-PPCDAM) and from 2010 through 2050 (post-PPCDAM).**
(ZIP)

Supporting Information S3 **Video showing cumulative deforestation probability from the pre-PPCDAM and post-PPCDAM scenarios, from 2002 to 2050 in the Brazilian Amazon, where from green to red means less to higher deforestation probability.**
(AVI)
